# Cortical hemodynamic responses in older stroke patients during a walking-based Stroop dual task: a functional near-infrared spectroscopy study

**DOI:** 10.3389/fnagi.2026.1714618

**Published:** 2026-02-11

**Authors:** Xiaohan Li, Kai Tang, Yuting Zhang, Lifeng Tang, Kun Wei, Lifeng Zhou, Min Tang

**Affiliations:** 1 Department of Neurological Rehabilitation, Ningbo Rehabilitation Hospital, Ningbo, China; 2 Department of Physical Education, Shenyang University of Chemical Technology, Shenyang, China; 3 Faculty of Marine, Ningbo University, Ningbo, China; 4 Faculty of Health Service and Health, Ningbo College of Health Sciences, Ningbo, China

**Keywords:** dual-task cost, functional near-infrared spectroscopy, gaitperformance, stroke, Stroop task

## Abstract

**Objectives:**

This study aimed to investigate cortical hemodynamic responses in older stroke patients during a walking-based Stroop dual task using functional near-infrared spectroscopy (fNIRS).

**Methods:**

Thirty-four stroke patients aged over 55 years performed three walking-based Stroop dual tasks while undergoing fNIRS recording: (a) color naming only (congruent task, CT), (b) color naming without color cues (neutral task, NT), and (c) incongruent color–word Stroop naming (incongruent task, IT). Gait parameters (walking speed, step length, stride width) and dual-task performance were measured. fNIRS data were analyzed to quantify cortical activation and functional connectivity (FC), focusing on the left and right prefrontal cortices (LPFC and RPFC) and motor cortices (LMC and RMC).

**Results:**

fNIRS revealed greater activation in the RPFC and bilateral motor cortices during the IT compared with the CT. Overall FC strength increased in the NT and IT, particularly between the LPFC and RPFC, LPFC and RMC, and RPFC and LMC. Walking speed and step length were significantly reduced in the IT compared with the CT, and dual-task performance declined under both higher-load conditions.

**Conclusion:**

Increasing Stroop-related cognitive demands during walking elicited greater prefrontal–motor activation and connectivity, accompanied by measurable dual-task costs in gait and performance. Slightly negative HbO values in the motor cortices during the simplest condition are likely driven by residual systemic influences rather than true deactivation, and our main inferences rely on within-subject contrasts across conditions. These findings indicate heightened cognitive–motor interference in older stroke survivors and highlight conflict processing as a key mechanism that may inform targeted dual-task rehabilitation strategies.

## 1 Introduction

Stroke is one of the leading causes of long-term disability among older adults worldwide, and individuals aged 55 years and above are at a substantially higher risk of both stroke and persistent post-stroke impairments (Mendis, 2013; West et al., 2014). This age group accounts for more than 70% of global stroke cases and is particularly prone to motor and cognitive deficits that markedly diminish functional independence and quality of life (Mendis, 2013; West et al., 2014). Among these deficits, impaired gait (e.g., reduced speed, unstable balance) and executive dysfunction (e.g., poor inhibitory control, attentional deficits) are particularly prevalent (Song et al., 2023). Importantly, these impairments rarely occur in isolation. Everyday community ambulation requires continuous integration of postural control, obstacle negotiation, and goal-directed cognition (Lim et al., 2022; Liu et al., 2018; Wang et al., 2023; Yang et al., 2018); thus, stroke-related damage often disrupts cognitive–motor coupling, resulting in cognitive–motor interference (CMI) (Wang et al., 2015).

Dual-task walking paradigms, in which individuals perform a cognitive task while walking, have therefore been widely used to investigate the interaction between motor and cognitive processes in stroke survivors (Liu et al., 2018; Wang et al., 2023). Unlike single-task assessments, dual-task paradigms better mimic real-world conditions, where walking is rarely isolated but accompanied by cognitive demands such as conversing, navigating crowds, or recalling instructions. According to capacity-sharing and bottleneck models (Pashler et al., 2001; Wollesen and Voelcker-Rehage, 2014), reduced cognitive resources and impaired executive function force stroke survivors to reallocate attention between gait and cognition, resulting in performance decrements in one or both domains (Lim et al., 2022; Yang et al., 2018). When gait impairment and executive dysfunction co-occur, this competition is further exacerbated, potentially elevating fall risk and functional dependence beyond what would be expected from either deficit alone. Understanding this interaction is therefore essential for identifying mechanisms underlying post-stroke mobility limitations.

The Stroop task is a well-established paradigm for probing executive function, particularly inhibitory control and conflict processing (MacLeod, 1991). Traditional Stroop tasks require naming the ink color of color-words that may be congruent or incongruent with their semantic meaning (Heiberg et al., 2023; MacLeod, 1991; Wollesen et al., 2016). Resolving this conflict engages executive processes that are closely associated with gait adaptability and fall risk in older adults and stroke survivors (Chan and Tsang, 2017; Lin et al., 2022; Yu et al., 2021). Compared with working-memory or arithmetic-based dual tasks (e.g., n-back or serial subtraction) (Plummer et al., 2020), Stroop-type conflict imposes particularly strong demand on resolving interference between competing response tendencies, which may be especially vulnerable after stroke and directly compromise safe walking in distracting environments. Although prior studies have documented pronounced Stroop interference (slower responses, more errors, and altered prefrontal activation) in stroke patients (Chan and Tsang, 2017; Lin et al., 2022; Yu et al., 2021), most have used seated, screen-based formats with limited relevance to everyday mobility. To enhance ecological validity, the present study incorporated Stroop-like conflict into a walking task requiring participants to visually search for and step onto target color-word cards. While still not fully replicate real-world walking, this paradigm combines locomotion, spatial exploration, and interference resolution, providing a more realistic challenge to cognitive–motor integration than traditional seated versions.

Functional near-infrared spectroscopy (fNIRS) offers a unique opportunity to examine the neural mechanisms underlying dual-task walking because it is portable, non-invasive, and relatively robust to motion artifacts (Wang et al., 2023). By measuring changes in oxygenated hemoglobin (HbO) and deoxygenated hemoglobin (HbR), fNIRS allows quantification of cortical activation and functional connectivity (FC) during ambulatory tasks (Ding et al., 2024; Leff et al., 2011). Recent fNIRS studies in stroke survivors using dual-task walking paradigms with different cognitive loads—such as serial subtraction (Al-Yahya et al., 2016; Chatterjee et al., 2019; Mori et al., 2018), n-back (Hermand et al., 2019, 2020), and verbal tasks (Hawkins et al., 2018; Lim et al., 2022)—have shown that increasing cognitive demand leads to elevated prefrontal activation, reduced gait automaticity, and broader engagement of premotor, sensorimotor, and parietal regions. Notably, neural patterns vary widely across individuals: some stroke survivors show inefficient overactivation or limited capacity for further recruitment, whereas others display adaptive compensatory responses. Such differences cannot be captured by behavioral measures alone, underscoring the need to investigate cortical load, resource allocation, and network-level coordination during dual-task walking. From a rehabilitation perspective, fNIRS-based markers of cortical activation and FC may ultimately guide the development and refinement of individualized dual-task training protocols that target specific neural deficits underlying CMI.

To address these gaps, the present study employed a walking-based Stroop paradigm with three levels of cognitive demand—congruent, neutral, and incongruent—to examine cortical hemodynamics, FC, and dual-task performance in older stroke survivors. We hypothesized that (Mendis, 2013) greater Stroop interference would elicit increased activation in prefrontal and motor regions and stronger FC within and between these areas, and (West et al., 2014) these neural changes would correspond to measurable dual-task costs in gait and dual-task performance. By integrating neural and behavioral measures, this study aims to provide insight into the mechanisms of CMI after stroke and inform the development of targeted rehabilitation strategies.

## 2 Materials and methods

### 2.1 Participants

Individuals with stroke were recruited from Ningbo Rehabilitation Hospital in September 2024. Demographic and clinical data, including age, gender, stroke type, lesion side, and duration of hemiparesis, were collected through structured interviews and medical chart reviews. To be included in the study, participants had to meet the following criteria: (Mendis, 2013) single stroke that occurred at least 6 months prior to participation; (West et al., 2014) age over 55 years (this threshold was set because individuals ≥55 are at high risk of stroke and long-term motor/cognitive impairments; aging-related declines in this group can further affect cognitive–motor integration, making them highly relevant to rehabilitation needs); (Song et al., 2023) ability to walk independently without the use of assistive devices; (Liu et al., 2018) stable medical condition with no comorbid neurological disorders; (Wang et al., 2023) a Mini-Mental State Examination (MMSE) score greater than 24 (Cockrell and Folstein, 2002); and (Lim et al., 2022) right-handedness. Participants were excluded if they had: (Mendis, 2013) significant musculoskeletal or cardiopulmonary disorders that could impair mobility; (West et al., 2014) severe visual or auditory impairments; or (Song et al., 2023) contraindications for fNIRS testing (e.g., scalp lesions, open wounds).

A total of 34 stroke patients (14 women and 20 men; mean age: 65.3 ± 4.1 years) were enrolled in the study. The sample size was determined based on statistical power analysis (G*Power 3.1) indicating that at least 24 participants would be required to detect significant differences in key outcomes across task conditions (one-way repeated-measures ANOVA, *f* = 0.4, α = 0.05, power = 0.85), and this number is consistent with similar fNIRS studies on dual-task (Li et al., 2025; Nosaka et al., 2023). All participants provided written informed consent before data collection. The study protocol was approved by the Institutional Review Board of Ningbo Rehabilitation Hospital (Approval No. 2022-03-G2) and was conducted in accordance with the principles outlined in the Declaration of Helsinki (latest revision).

### 2.2 Design protocol

**FIGURE 1:**
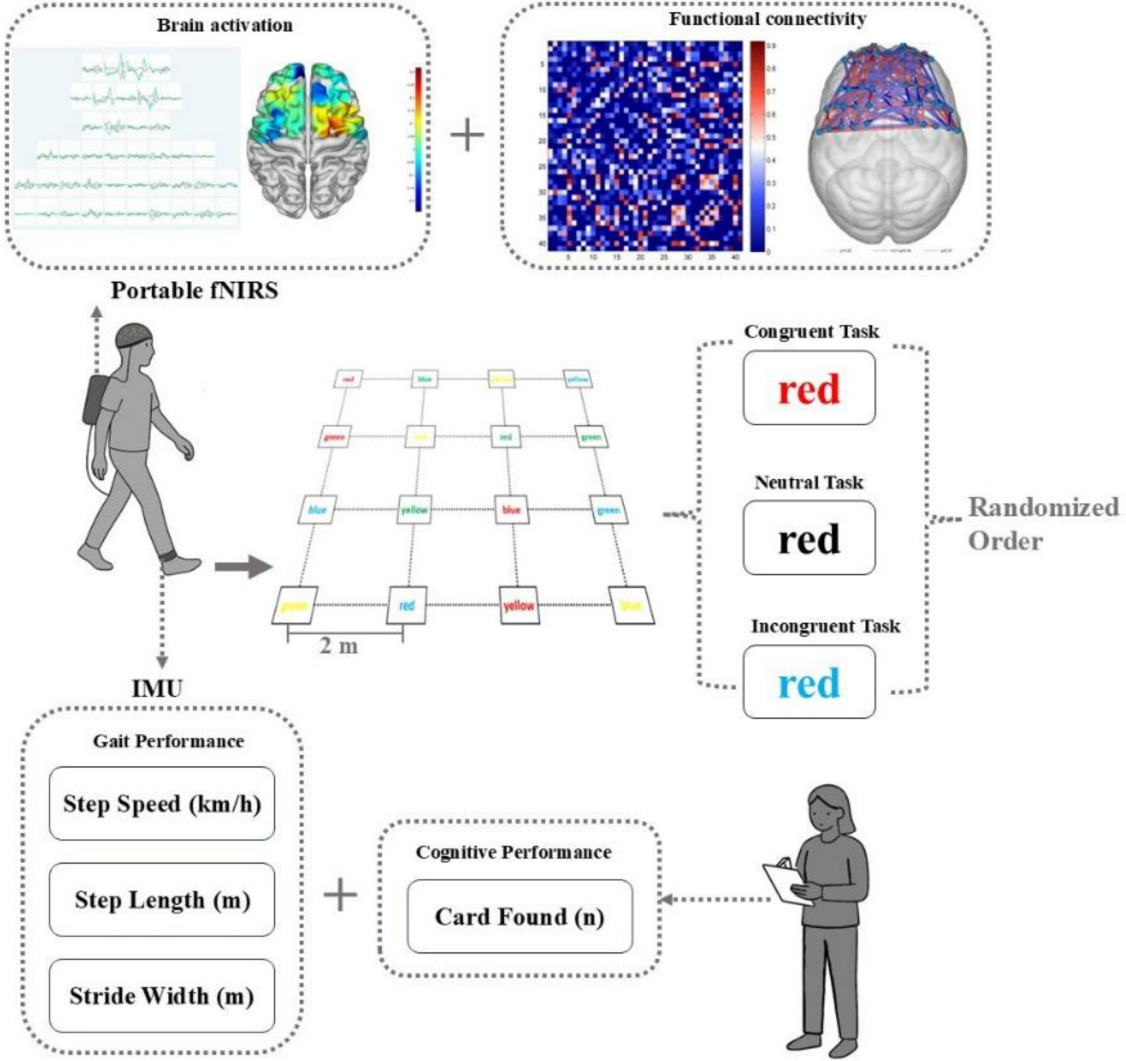
Experimental protocol.

This study employed a walking-based Stroop dual-task paradigm developed and validated by our research team in a prior study involving healthy adults, with the findings reported in a related publication (Li et al., 2025). Figure 1 illustrates the design protocol of this study.

Experimental Setup: A 6 × 6 m grid was marked on the floor of a quiet, well-lit laboratory. Within the grid, 4 × 4 matrix of color-word cards (20 cm × 20 cm) was placed, with each card spaced 2 m apart (center-to-center). The cards displayed color terms (“red,” “blue,” “green,” “yellow”) in four possible font colors (red, blue, green, yellow) or black (neutral condition).

Task conditions: Three difficulty levels were designed based on Stroop interference principles: (Mendis, 2013) congruent task (CT): congruent condition, where the printed word matched its font color (e.g., the word “red” printed in red ink); (West et al., 2014) neutral task (NT): black-font condition, where all words were printed in black ink, removing the color cue; (Song et al., 2023) incongruent task (IT): incongruent condition, where the printed word and its font color did not match (e.g., the word “red” printed in green ink), introducing Stroop-like interference.

Test procedure: Before testing, participants received standardized instructions and performed a short familiarization trial to ensure task comprehension. Each task commenced with the participant standing at the designated starting point (center of the grid). The researcher verbally announced the target, and the participant immediately began walking to locate the target card. Upon finding the correct card, they moved to its location and awaited the next instruction, continuing without returning to the starting point. Each task lasted 1 min, followed by a 1-min seated rest (timed using a stopwatch). The three tasks were presented in randomized order to minimize learning and order effects.

### 2.3 Gait and dual-task performance

Gait was measured using an inertial measurement unit (IMU; MTw Awinda, Xsens, Netherlands) attached to the medial malleolus of the non-hemiparetic leg using medical tape. The IMU recorded tri-axial acceleration and angular velocity and has an internal sampling rate of 1,000 Hz. Data were recorded and processed using Xsens MVN Analyze software (v2021.2). Based on the trajectory of the instrumented ankle, the following gait parameters were extracted: (Mendis, 2013) walking speed (km/h), calculated as the average forward velocity of the instrumented ankle over the walking period; (West et al., 2014) step length (m), defined as the anterior–posterior displacement of the instrumented (non-hemiparetic) ankle between successive heel strikes of that same limb, averaged across all steps, providing an estimate of average step size; and (Song et al., 2023) stride width (m), estimated as the medio–lateral distance between successive footprints inferred from the mediolateral trajectory of the instrumented ankle, averaged across all steps.

Dual-task performance during the walking Stroop conditions was assessed using the number of correctly identified target cards (“cards found”). During each trial, participants were instructed to walk through the grid and step onto cards that matched the task requirement, while an examiner recorded the total number of correctly selected targets. Because reaching each card required both accurate cognitive processing (e.g., visual search and conflict resolution) and locomotor behavior (e.g., walking speed and path efficiency), this measure reflects a composite index of cognitive–motor performance rather than a pure cognitive outcome.

### 2.4 fNIRS acquisition and analysis

**FIGURE 2:**
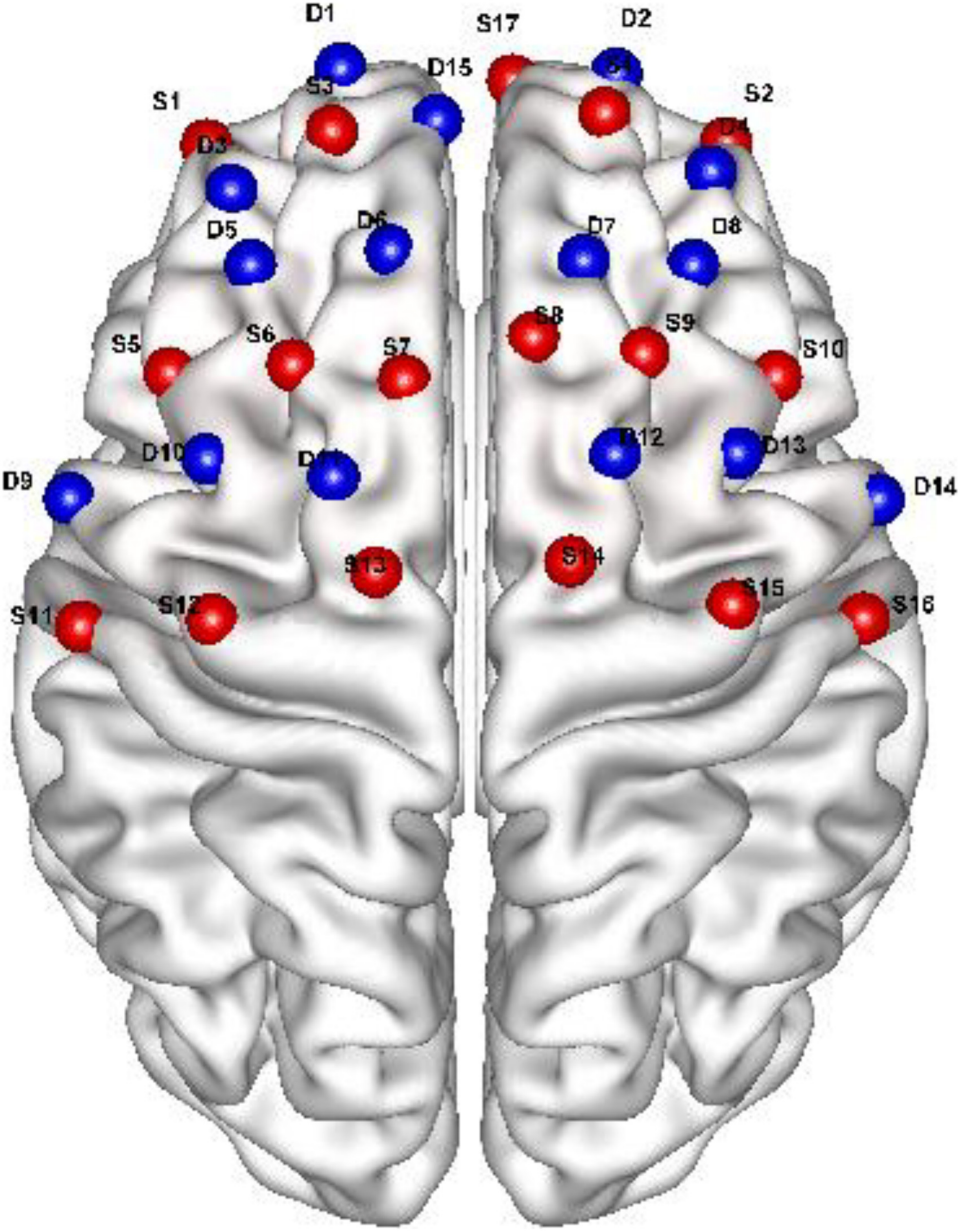
Schematic of functional near-infrared spectroscopy (fNIRS) channel arrange.

Cortical hemodynamic responses were recorded using a continuous-wave, wireless portable NirSmart 6000A fNIRS system (Danyang Huichuang Medical Equipment Co., China) with optodes positioned according to the international 10–20 system to cover left and right prefrontal cortices (LPFC and RPFC) and motor cortices (LMC and RMC). The setup consisted of 41 channels, formed by 17 source optodes and 15 detector optodes (Figure 2). Table 1 shows the Montreal Neurological Institute (MNI) coordinates. The system emitted near-infrared light at wavelengths of 760 and 850 nm, with a sampling rate of 11 Hz. The emitter-detector distance was 3 cm.

**TABLE 1:** The MNI coordinates and HbO of channels.

CH (S-D)	MNI			ROI	HbO (mmol/L × mm)			ANOVA	
	x	y	z		CT	NT	IT	F	*P*
CH1 (S1-D1)	−33.6667	65.3333	2.3333	LPFC	0.037 ± 0.033	0.069 ± 0.097	−0.085 ± 0.176	2.330	0.060
CH2 (S1-D3)	−37.6667	59.6667	16.3333	LPFC	−0.023 ± 0.094	0.042 ± 0.081	0.013 ± 0.057	1.159	0.099
CH3 (S2-D2)	31.6667	68	1.6667	RPFC	−0.136 ± 0.250	0.045 ± 0.314^a^	0.109 ± 0.095^a^	3.186	0.036
CH4 (S2-D4)	39.6667	61	14	RPFC	0.055 ± 0.053	−0.163 ± 0.345^a^	−0.018 ± 0.059^a^	3.051	0.038
CH5 (S3-D1)	−19.6667	70	16.6667	LPFC	−0.040 ± 0.056	0.003 ± 0.057	0.054 ± 0.094	2.163	0.067
CH6 (S3-D3)	−27.3333	59	27.3333	LPFC	−0.006 ± 0.152	−0.029 ± 0.048	−0.019 ± 0.064	0.089	0.520
CH7 (S3-D15)	−11.3333	65.3333	29.3333	LPFC	−0.062 ± 0.100	0.092 ± 0.111^a^	−0.065 ± 0.103^b^	4.964	0.016
CH8 (S4-D2)	21.6667	71	15.6667	RPFC	0.011 ± 0.094	0.073 ± 0.121	−0.021 ± 0.271	0.422	0.418
CH9 (S4-D4)	28.6667	62.3333	25.6667	RPFC	0.160 ± 0.185	−0.049 ± 0.285	0.026 ± 0.142	1.683	0.082
CH10 (S4-D15)	12.6667	67	28.6667	RPFC	−0.042 ± 0.270	−0.160 ± 0.214^a^	−0.023 ± 0.187^a^	2.868	0.040
CH11 (S5-D5)	−41.3333	33.3333	43.3333	LMC	−0.020 ± 0.124	0.056 ± 0.080	0.114 ± 0.089^a,b^	3.561	0.035
CH12 (S5-D9)	−54	15.3333	37.3333	LMC	−0.066 ± 0.165	−0.013 ± 0.161^a^	0.061 ± 0.079^a^	2.807	0.044
CH13 (S5-D10)	−46.3333	16.3333	51.6667	LMC	−0.077 ± 0.142	−0.034 ± 0.053^a^	0.048 ± 0.078^a,b^	3.504	0.035
CH14 (S6-D5)	−33.3333	33.6667	49.3333	LMC	−0.117 ± 0.306	0.056 ± 0.132	−0.035 ± 0.080	1.377	0.084
CH15 (S6-D6)	−18.6667	35.6667	57	LMC	−0.065 ± 0.062	−0.139 ± 0.268	0.056 ± 0.120^a^	2.767	0.046
CH16 (S6-D10)	−38.6667	17.6667	59	LMC	0.015 ± 0.070	−0.016 ± 0.398	0.018 ± 0.250	0.033	0.562
CH17 (S6-D11)	−26.3333	17.3333	65	LMC	0.102 ± 0.358	0.017 ± 0.246	−0.015 ± 0.036	0.489	0.356
CH18 (S7-D6)	−13.6667	35.6667	59	LMC	0.009 ± 0.053	0.076 ± 0.157	0.132 ± 0.265	0.730	0.332
CH19 (S7-D11)	−16	18	68.6667	LMC	0.102 ± 0.159	0.150 ± 0.409	−0.025 ± 0.124	1.081	0.139
CH20 (S8-D7)	14.3333	37.6667	58.3333	RMC	−0.039 ± 0.114	0.007 ± 0.088	0.062 ± 0.167	1.096	0.138
CH21 (S8-D12)	18	17.3333	68.6667	RMC	0.267 ± 0.400	−0.017 ± 0.062	0.067 ± 0.116	1.204	0.089
CH22 (S9-D7)	24.3333	37	55.3333	RMC	0.032 ± 0.134	0.047 ± 0.249	−0.020 ± 0.167	0.290	0.470
CH23 (S9-D8)	34.3333	35.6667	49.6667	RMC	−0.098 ± 0.178	0.046 ± 0.110^a^	0.013 ± 0.034^a^	2.813	0.043
CH24 (S9-D12)	29.6667	21.6667	62	RMC	−0.045 ± 0.102	−0.090 ± 0.097^a^	0.050 ± 0.094^a,b^	4.281	0.027
CH25 (S9-D13)	40.6667	18.3333	58.3333	RMC	−0.050 ± 0.054	0.021 ± 0.081	0.027 ± 0.159	0.907	0.2763
CH26 (S10-D8)	44.6667	34.6667	41.6667	RMC	−0.054 ± 0.134	−0.148 ± 0.389	0.020 ± 0.137	0.960	0.208
CH27 (S10-D13)	49	18.6667	49.6667	RMC	0.055 ± 0.186	−0.053 ± 0.125^a^	0.104 ± 0.131	2.705	0.047
CH28 (S10-D14)	57	14.6667	36.6667	RMC	0.058 ± 0.081	0.039 ± 0.054	0.132 ± 0.101	2.153	0.078
CH29 (S11-D9)	−61	−5	41	LMC	0.011 ± 0.059	0.043 ± 0.087	0.071 ± 0.128	0.672	0.342
CH30 (S11-D10)	−52.3333	−4.3333	54.6667	LMC	0.057 ± 0.104	−0.005 ± 0.085	0.026 ± 0.134	0.462	0.398
CH31 (S12-D10)	−42.6667	−1.6667	61.3333	LMC	−0.071 ± 0.105	−0.033 ± 0.062	−0.026 ± 0.080	0.591	0.351
CH32 (S12-D11)	−30	0	68	LMC	−0.081 ± 0.156	0.019 ± 0.127	−0.043 ± 0.090	1.060	0.191
CH33 (S13-D11)	−18.3333	2.3333	73.3333	LMC	−0.049 ± 0.055	0.275 ± 0.748	0.007 ± 0.065^a^	2.572	0.049
CH34 (S14-D12)	19.6667	2.3333	73.6667	RMC	−0.122 ± 0.126	0.008 ± 0.080	−0.025 ± 0.094	0.780	0.319
CH35 (S15-D12)	31.6667	0.66667	67.3333	RMC	−0.048 ± 0.096	−0.276 ± 0.569^a^	0.302 ± 0.371^a,b^	4.305	0.026
CH36 (S15-D13)	44.6667	1	61	RMC	−0.085 ± 0.100	0.213 ± 0.195	0.188 ± 0.164^a,b^	7.058	0.004
CH37 (S16-D13)	55.6667	−1.6667	52.6667	RMC	−0.018 ± 0.202	−0.595 ± 0.926	−0.002 ± 0.215	1.783	0.081
CH38 (S16-D14)	64.6667	−4.3333	39.3333	RMC	−0.050 ± 0.102	−0.154 ± 0.165	−0.122 ± 0.250	0.468	0.363
CH39 (S17-D1)	−14.6667	73.3333	4.3333	LPFC	−0.050 ± 0.090	−0.051 ± 0.033	−0.028 ± 0.087	0.226	0.493
CH40 (S17-D2)	13.3333	74	3.3333	RPFC	−0.013 ± 0.119	0.098 ± 0.150	0.130 ± 0.236	1.127	0.120
CH41 (S17-D15)	0.66667	67	16.3333	LPFC	−0.022 ± 0.047	−0.022 ± 0.103	−0.027 ± 0.097	0.010	0.619

The superscript letters indicate *post-hoc* pairwise comparisons. Specifically, “a” denotes a significant difference compared with the CT condition (*P* < 0.05), and “b” denotes a significant difference compared with the NT condition (*P* < 0.05). CH, channel; CT, congruent task; HbO, oxygenated; IT, incongruent task; LMC, left motor cortex; LPFC, left prefrontal cortex; MNI, Montreal neurological institute; NT, neutral task; RMC, right motor cortex; ROI, regions of interest; RPFC, right prefrontal cortex.

Raw optical density data were preprocessed using NirSpark software (Danyang Huichuang Medical Equipment Co., Ltd., China). Motion artifacts were corrected with spline interpolation (standard deviation threshold = 6, peak threshold = 0.5). The signals were converted into optical density and band-pass filtered at 0.01–0.2 Hz to remove physiological noise (respiration, cardiac pulsation, Mayer waves). Hemoglobin concentration changes in HbO and HbR forms were calculated using the modified Beer–Lambert law (differential pathlength factor = −6 to 6).

For each trial, signals were baseline-corrected using the 10-s seated resting period immediately prior to task onset. Given that the walking-based Stroop paradigm required repeated transitions between gait initiation, brief stopping, and continued walking, the task-related regressors in the general linear model (GLM) were time-locked to the entire 1-min task period, thereby minimizing the influence of transient gait-initiation responses on the estimation of sustained cortical activation. Although these procedures reduce the impact of systemic hemodynamic fluctuations associated with movement onset and offset, residual contamination from global blood pressure and heart rate changes cannot be fully excluded. Given its higher sensitivity to cerebral blood flow changes, HbO was adopted as the primary indicator (Aleksandrowicz et al., 2020; Li et al., 2025; Teo et al., 2021). A GLM was applied to fit the hemodynamic response function (HRF), with the resulting beta (β) values reflecting task-related cortical activation. FC was assessed by calculating Pearson correlation coefficients of HbO time series across channels and regions of interest (ROI), followed by Fisher’s Z transformation. Multiple comparisons were corrected using the false discovery rate (FDR) method.

### 2.5 Statistical analysis

All statistical analyses were conducted using SPSS version 27.0 (IBM Corp., Armonk, NY, United States). The Shapiro–Wilk test was used to examine data normality, and Levene’s test was applied to verify homogeneity of variances. Descriptive data are presented as mean ± standard deviation (SD). A one-way repeated-measures ANOVA was performed to compare gait parameters, cognitive performance, and fNIRS-derived hemodynamic responses across the three task conditions (CT, NT, and IT). *Post hoc* comparisons were corrected using the Bonferroni method. Statistical significance was defined as *P* < 0.05.

## 3 Results

### 3.1 Results of brain activation

**FIGURE 3:**
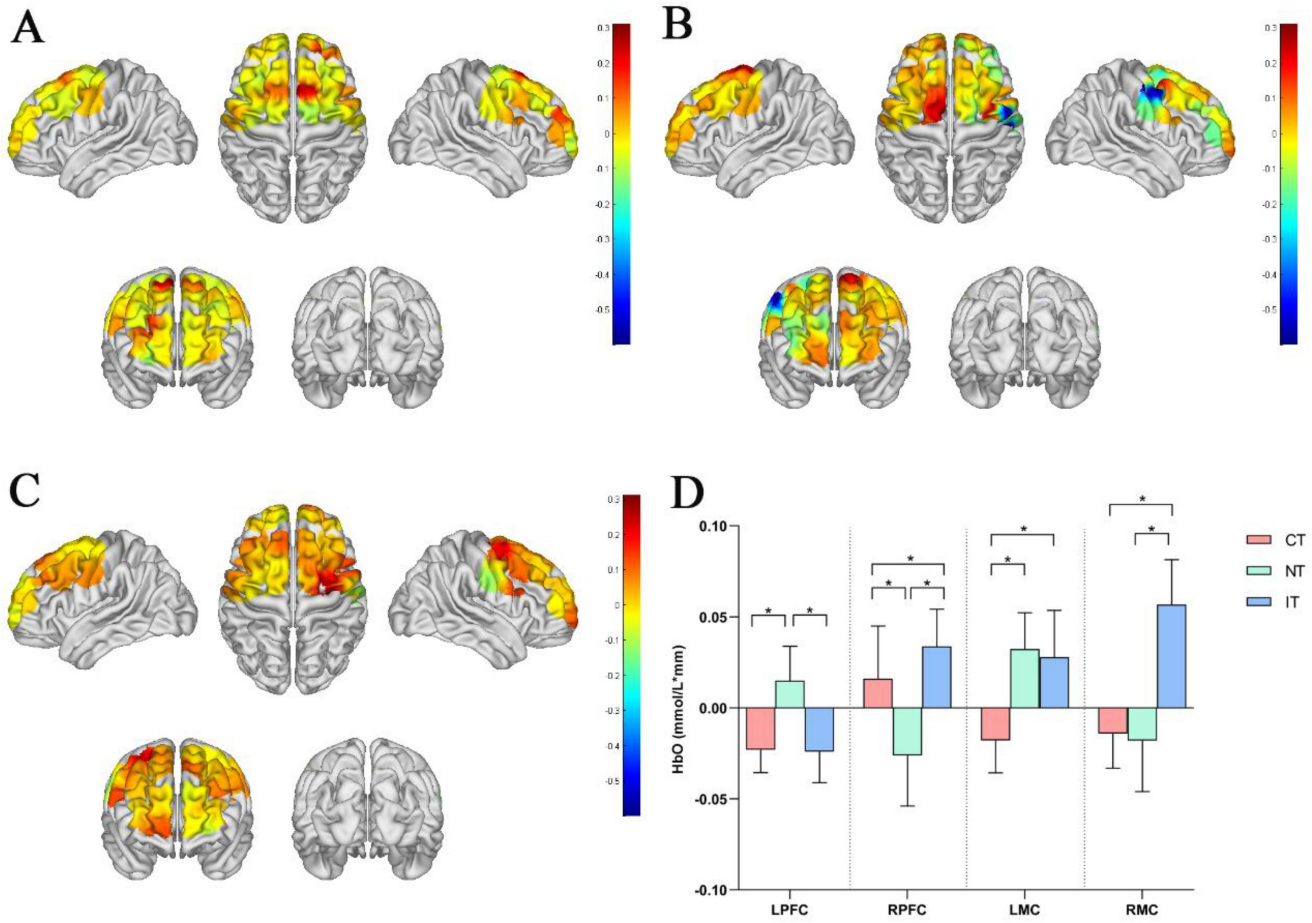
Results of brain activation. **(A)** Cortical activation map under CT, **(B)** cortical activation map under NT, **(C)** cortical activation map under IT, **(D)** comparison of cortical activation based on regions of interest (ROI) under three task conditions. CT, congruent task; IT, incongruent task; LMC, left motor cortex; LPFC, left prefrontal cortex; NT, neutral task; RMC, right motor cortex; RPFC, right prefrontal cortex; *P < 0.05.

Figure 3A illustrates the cortical activation map under the CT condition, Figure 3B under the NT condition, and Figure 3C under the IT condition. Figure 3D presents the ROI-based comparisons of cortical activation across the three task conditions. The results revealed that in the LPFC, HbO concentration during NT (0.015 ± 0.019) was significantly higher than during CT (−0.023 ± 0.012) and IT (−0.024 ± 0.017). In the RPFC, HbO concentration during IT (0.034 ± 0.020) was significantly higher than during both CT (0.016 ± 0.029) and NT (−0.026 ± 0.027), and CT also showed greater activation compared to NT. In the LMC, HbO concentration was significantly higher during NT (0.032 ± 0.020) and IT (0.027 ± 0.025) compared to CT (−0.017 ± 0.016). In the RMC, HbO concentration during IT (0.056 ± 0.024) was significantly higher than during CT (−0.014 ± 0.019) and NT (−0.018 ± 0.028). Further channel-level analysis indicated significant differences in HbO concentration across tasks at channels 3, 4, 7, 10, 11, 12, 13, 15, 23, 24, 27, 33, 35, and 36 (Table 1).

### 3.2 Results of functional connectivity

**FIGURE 4:**
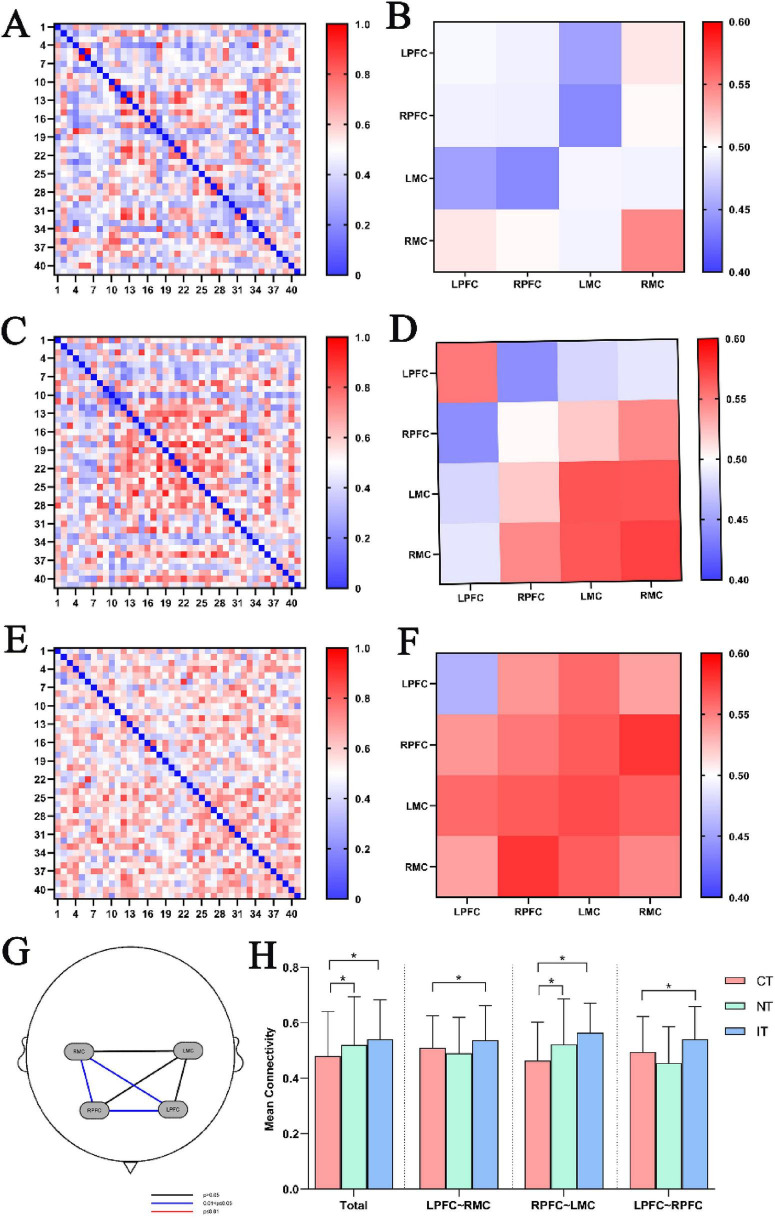
Results of functional connectivity. **(A)** Channel-based FC under CT, **(B)** regions of interest (ROI)-based FC under CT, **(C)** channel-based FC under NT, **(D)** ROI-based FC under NT, **(E)** channel-based FC under IT, **(F)** ROI-based FC under IT, **(G)** comparison of FC strength based on ROI under three task conditions, **(H)** total, LPFC-RMC, RPFC-LMC and LPFC-RPFC FC under three task conditions. CT, congruent task; FC, functional connectivity; IT, incongruent task; LMC, left motor cortex; LPFC, left prefrontal cortex; NT, neutral task; RMC, right motor cortex; RPFC, right prefrontal cortex; *P < 0.05.

Figures 4A–F illustrate the channel- and ROI-based FC patterns of participants across the three task conditions. Figure 4H demonstrates that overall FC strength was significantly higher during NT (0.520 ± 0.174) and IT (0.540 ± 0.143) compared to CT (0.480 ± 0.161). Further ROI-to-ROI analyses revealed significant task-related differences in connectivity strength among LPFC-RMC, RPFC-LMC, and LPFC-RPFC (Figure 4G). Specifically, LPFC-RMC connectivity strength was significantly greater during IT (0.536 ± 0.125) compared to CT (0.509 ± 0.116); RPFC-LMC connectivity strength was significantly greater during NT (0.521 ± 0.164) and IT (0.564 ± 0.107) compared to CT (0.462 ± 0.139); and LPFC-RPFC connectivity strength was significantly greater during IT (0.541 ± 0.118) compared to CT (0.493 ± 0.129) (Figure 4H).

### 3.3 Results of gait and dual-task performance

**FIGURE 5:**
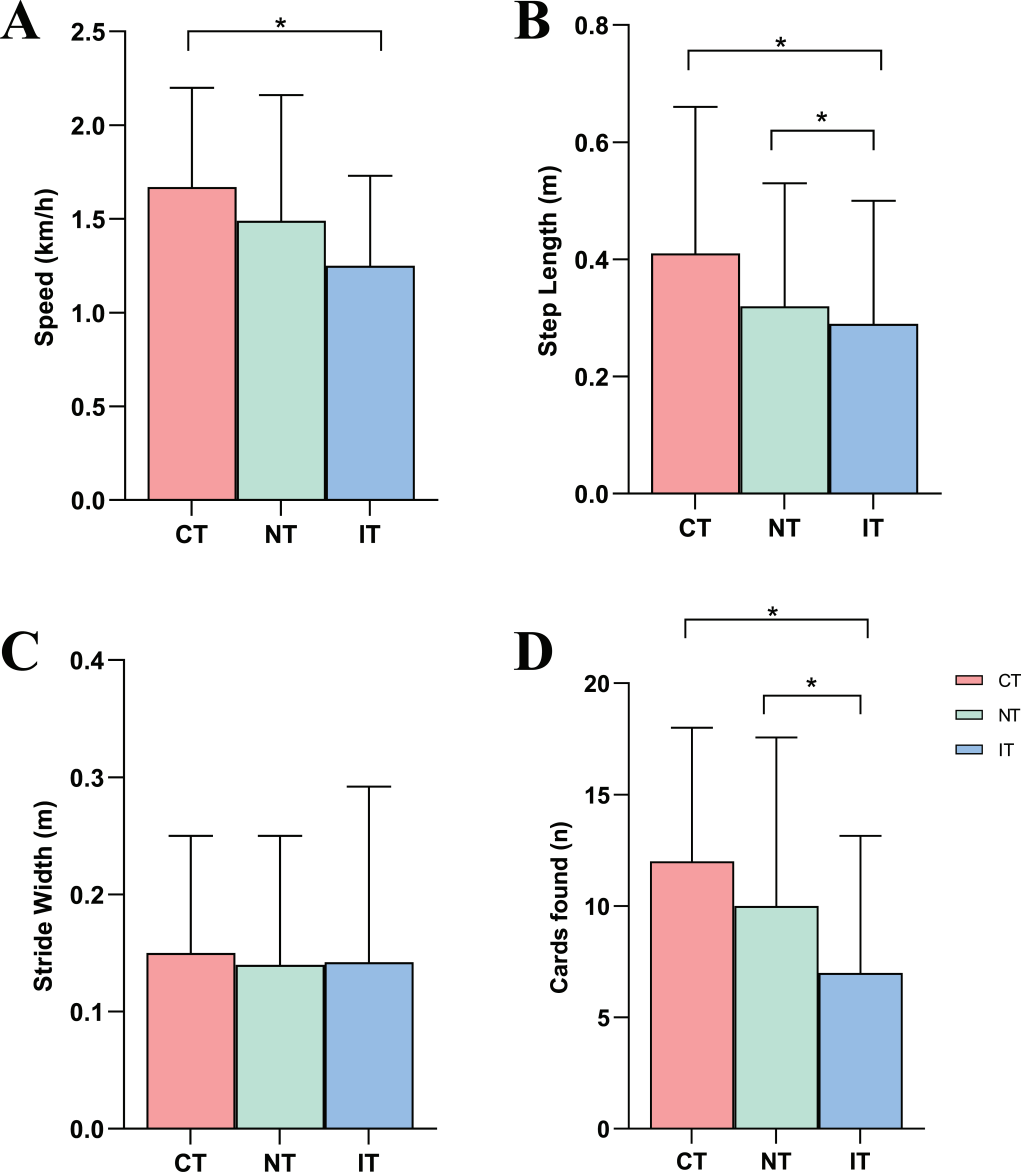
Results of gait and cognitive performances. **(A)** Speed, **(B)** step length, **(C)** stride width, **(D)** cards found. CT, congruent task; IT, incongruent task; NT, neutral task; *P < 0.05.

Figure 5 presents the results of gait and dual-task performance across the three task conditions. For gait parameters, walking speed was significantly lower during IT (1.25 ± 0.48) compared to CT (1.67 ± 0.53), while step length was significantly reduced during both NT (0.32 ± 0.21) and IT (0.29 ± 0.21) relative to CT (0.41 ± 0.25). No significant differences were observed in stride width across the three conditions. Regarding dual-task performance, the number of correctly identified cards was significantly lower during NT (10.00 ± 7.56) and IT (7.00 ± 6.15) compared to CT (12.00 ± 6.00). Given that walking speed was also reduced under the higher-load conditions, especially IT, these differences in “cards found” should be interpreted as reflecting the combined impact of increased cognitive demand and slower locomotion, rather than a pure index of cognitive processing efficiency.

## 4 Discussion

### 4.1 Discussion of brain activation

The present results partly support the hypothesis that increasing task difficulty enhances activation in both the prefrontal and motor cortices, while also revealing region-specific patterns of engagement. In the LPFC, cortical activation was significantly greater during the NT compared with both the CT and IT tasks. This pattern suggests that, when color cues were removed, stroke patients relied more heavily on LPFC-mediated attentional control to sustain task performance. In contrast, the RPFC exhibited significantly stronger activation during the IT relative to both CT and NT. Given the RPFC’s established role in suppressing task-irrelevant information and resolving interference (Laguë-Beauvais et al., 2013; Xiang et al., 2023), this heightened activation likely reflects the increased need for conflict monitoring and inhibitory control under IT. The observed asymmetry between the LPFC and RPFC therefore indicates that stroke patients dynamically recruit lateralized prefrontal networks depending on task characteristics-engaging the LPFC to manage ambiguous, cue-poor contexts and the RPFC to cope with high levels of conflict.

For the motor cortices, both left and right hemispheres demonstrated significantly greater activation during IT compared with CT. The finding is consistent with our previous study in healthy adults, where incongruent Stroop walking also elicited enhanced motor cortex activation (Li et al., 2025). This aligns with the notion that increasing cognitive load amplifies demands on motor control during walking, as CMI requires greater sensorimotor integration and postural adjustments to maintain gait stability (Aldraiwiesh, 2024; Bayot et al., 2018; Small et al., 2021). At the channel level, widespread differences across prefrontal and motor areas further support the view that Stroop dual-task walking imposes complex, distributed neural demands in stroke patients.

An important methodological issue concerns the slightly negative HbO values observed in the motor cortices during CT. Because walking necessarily requires activation of lower-limb motor regions, true “deactivation” below a seated baseline is unlikely. These small negative values are more plausibly explained by residual systemic hemodynamic fluctuations—such as gait initiation, transient blood pressure and heart-rate changes, and global scalp blood-flow shifts—that may not have been fully removed by standard band-pass filtering and spline interpolation, as well as by the use of a brief 10-s seated baseline that is vulnerable to drift. Consequently, motor cortex activation during CT was likely underestimated. Our main conclusions, however, are based on relative differences between conditions: activation in the motor cortices increased from CT to the higher-load NT and IT tasks, and these within-subject contrasts are less affected by baseline bias. Nonetheless, the presence of negative HbO values calls for caution when interpreting absolute activation levels and highlights the need for methodological refinements in future work, such as short-separation channels, physiological regressors (e.g., accelerometry, heart rate), and longer or standing baselines to better separate cortical signals from systemic influences during ambulatory fNIRS.

### 4.2 Discussion of functional connectivity

Overall FC strength was significantly higher during the NT and IT conditions compared with the CT condition, indicating that as task difficulty increases, the brain requires more complex cognitive resources and motor regulation, necessitating enhanced information transfer and coordination among ROIs to achieve efficient integration of cognitive control and motor execution. Under the CT condition, cognitive conflict is minimal and only basic network coupling is necessary to perform the task, resulting in the lowest FC strength. In contrast, when color cues are removed (NT) or when semantic-color conflict peaks (IT), the brain must further elevate overall FC to ensure simultaneous conflict inhibition, spatial orientation, and gait regulation-a pattern that has also been reported in healthy adults (Huang et al., 2022; Zhang et al., 2013).

As task demands increase, the pattern of ROI-to-ROI connectivity exhibits targeted reorganization. In the IT condition, connectivity between the bilateral prefrontal cortices (LPFC-RPFC) and between each prefrontal region and its contralateral motor cortex (LPFC-RMC, RPFC-LMC) was significantly stronger than in the CT condition. The strengthened LPFC-RPFC coupling suggests that stroke patients integrate the functional advantages of both hemispheres: the LPFC supports focused attention on font color and suppression of automatic word reading, while the RPFC facilitates conflict detection and resolution (Anderson et al., 2025; He et al., 2025; Lu et al., 2025). Likewise, the enhanced LPFC-RMC and RPFC-LMC connections indicate that higher task difficulty requires tighter communication between cognitive control centers and motor execution regions to synchronize executive decision-making with gait adjustments.

### 4.3 Discussion of Gait and dual-task performances

The present study showed clear dual-task effects on gait performance in older stroke survivors. Walking speed and step length decreased progressively from the CT to the IT Stroop conditions, indicating that increasing cognitive demand imposed additional constraints on locomotor control. This pattern aligns with capacity-sharing models of CMI, which posit that stroke-related reductions in attentional and executive resources can limit the ability to maintain stable gait when cognitive demands rise (Pashler et al., 2001; Wollesen and Voelcker-Rehage, 2014). Interestingly, stride width remained stable across all conditions, implying that participants prioritized maintaining lateral stability a commonly observed compensatory strategy among individuals with balance impairments to minimize fall risk (Small et al., 2021).

Dual-task performance, indexed by the number of correctly identified target cards, also declined as cognitive load increased. However, because participants walked more slowly in the IT condition, the lower number of cards found likely reflects the combined impact of slower locomotion and greater cognitive demand, rather than a purely cognitive decline. This supports interpreting “cards found” as a composite cognitive–motor performance measure, influenced by both visual search and movement through the grid. Overall, these findings indicate that increased conflict-related demands during walking exacerbate CMI in stroke survivors, leading to measurable changes in both gait control and task performance and highlighting the need to assess cognitive and motor systems together when evaluating functional status and fall risk.

### 4.4 Clinical implications and limitations

From a clinical perspective, these findings have several important implications for the assessment and rehabilitation of older stroke patients. First, the marked over-activation of prefrontal and motor cortices and the strengthened FC observed under relatively brief, ecologically relevant dual-task conditions suggest that everyday situations requiring walking while processing conflicting information (e.g., navigating busy environments or responding to distracting cues) may place a disproportionate burden on already compromised neural systems. This underscores the need to routinely evaluate dual-task walking, rather than relying solely on single-task gait tests or simple cognitive assessments, when estimating fall risk and functional capacity in stroke survivors. Second, the distinct patterns of LPFC- and RPFC-dominant engagement, together with increased cross-hemispheric prefrontal–motor coupling under higher Stroop demands, point to potential neural targets for intervention. Training protocols that incorporate conflict-rich, Stroop-like dual tasks during walking may help patients practice reallocating executive resources while maintaining gait stability. In addition, integrating behavioral dual-task costs with fNIRS-derived markers of cortical activation and FC may facilitate individualized rehabilitation by identifying patients with limited compensatory reserve who require more gradual progression of dual-task training and closer supervision in complex walking environments.

Several limitations should be acknowledged. First, the sample size was modest, and the absence of an age-matched healthy control group limits the ability to distinguish stroke-specific effects from age-related changes. Second, although fNIRS is suitable for ambulatory neuroimaging, it remains susceptible to systemic physiological noise. The slightly negative HbO values observed in the motor cortices during the CT condition suggest residual contamination from blood pressure fluctuations, heart-rate changes, or baseline drift; absolute activation levels should therefore be interpreted with caution. Third, gait performance was derived from a single ankle-mounted IMU, which provides reliable step-based metrics but cannot fully characterize bilateral gait dynamics. Fourth, the “cards found” measure reflects a composite of cognitive and locomotor behavior and is influenced by walking speed; although this corresponds to our conceptualization of dual-task performance, it does not isolate cognitive processing. Finally, the walking Stroop task—while more naturalistic than seated versions—remains a laboratory-designed paradigm that does not fully replicate real-world mobility contexts. Future studies incorporating larger samples, complementary motion-capture systems, short-separation channels, and more ecological dual-task environments will help refine these findings and strengthen their translational value for clinical practice.

## 5 Conclusion

This study shows that increasing Stroop-related cognitive demands during walking leads to greater activation in prefrontal and motor cortices, stronger prefrontal–motor connectivity, and clear dual-task costs in gait and performance in older stroke survivors. Slightly negative HbO values in the motor cortices during the simplest condition are likely driven by residual systemic influences rather than true deactivation, and our main inferences rely on within-subject contrasts across conditions. Overall, the combined behavioral and fNIRS findings highlight conflict processing as a key contributor to CMI and a potential target for individualized dual-task rehabilitation.

## Data Availability

The original contributions presented in this study are included in this article/supplementary material, further inquiries can be directed to the corresponding author.
